# Irrational polypharmacy and potentially inappropriate medications in patients with dementia: Treatment strategies

**DOI:** 10.1192/j.eurpsy.2023.105

**Published:** 2023-07-19

**Authors:** M. Štuhec, EPA2023SP-1103

**Affiliations:** Department for Pharmacology, University of Maribor, Medical Faculty & Ormoz Psychiatric Hospital, Slovenia, Maribor, Slovenia

## Abstract

**Abstract:**

Most patients with dementia are treated with irrational polypharmacy, which leads to higher mortality and other negative consequences (higher costs). Nearly 50% of elderly patients take one or more medications that are not medically necessary, which represents another important aspect of medication optimization. Research has established a strong relationship between irrational polypharmacy and its negative clinical consequences, including a negative impact on dementia, especially in patients with excessive polypharmacy (10 or more medications). These patients are underrepresented in the treatment guidelines and randomized controlled trials, although they represent a substantial patient population. The burden of irrational polypharmacy has also been associated with a greater risk of adverse drug events, drug-drug interactions, medication non-adherence and a higher risk of potentially inappropriate medication (PIM) use. Different treatment strategies have been available to reduce irrational polypharmacy in this population. The best intervention for irrational polypharmacy reduction in this population involves an inter-professional approach (collaborative care approach) that often includes special tools, a basic pharmacological approach and collaboration with a clinical pharmacist.

**Disclosure of Interest:**

None Declared

